# Book Reviews and Notices

**Published:** 1886-08

**Authors:** 


### BIBLIOGRAPHY.

DISEASES OF THE SPINAL CORD, by Byron Bramwell, M. D., F. R. C. P., (Edin), lecturer on Principles and Practice of Medicine and on Medical Diagnosis in the Extra Academical School of Medicine, Edinburgh · and ever so many other titles which we think it unnessary to enumerate. With fifty-three colored plates and two fine wood engravings. Second edition, New York, Wm. Wood & Co., 1886.

This is the January number of "Wood's Standard Library" for 1886. It is dedicated to Prof. Charcot, of Paris, and Prof. Erb, of Leipzig, to whom the author says he is indebted for his knowledge of the subject.

An important feature of this work, and one which is the outgrowth of modern civilization,—is the chapter on concussion of the spine and the method of examining in "railroad cases." No doubt, railroad corporations have been swindled out of millions for alleged damages to the spinal cord. Those cases of injury "where there is no lesion, no solution of continuity and which are laid to "shock" or "concussion," form a large per cent. of the claims for damages by railway accidents; and in the interest of justice as well as of the rights of railroad corporations, it is important to be able to say if there be real injury to the spine, and to what extent,—or if the claimant is only "malinguering." This book is a valuable addition to the literature on the subject of spinal diseases and injuries.

Gotten out in uniform style, maroon cloth and gilt, with the other volumes of the "Library" for 1886.

---

HAND-BOOK OF PRACTICAL MEDICINE. By Herman Eichorst, Professor of Special Pathology and Therapeutics, Director of the University Medical Clinic in Zurich, Vol. I, *Diseases of the Circulatory and Respiratory Apparatus.* One hundred and three wood engravings. New York: William Wood & Co. 1886.

This is the March number of Wood's library of standard medical authors, uniform with the series for 1886.

---

THE GENUINE WORKS OF HIPPOCRATES. Translated from the Greek, with a preliminary discourse and annotations. By Francis Adams, LL. D., Surgeon. In two volumes. Vol. I, New York: Wm. Wood & Co. 1886. This is the April number of Wood's library.

To those who find pleasure in poring over the theories and speculations of the *Father*, and in instituting comparisons, and estimating the progress of medicine since that day, this work will be interesting, There being so much doubt and obscurity on the subject of Hippocrates' writings, the Sydenham Society requested Mr. Adams to thoroughly investigate the works bearing his name, and to cull out of them those that are known to be genuine, and to furnish a translation. Messrs. Wood & Co. have presented here the first volume of the labors of the author; and, as it bears the seal of approval of the Sydenham Society, we suppose there is no doubt as to its being genuine. Every student of medicine should be familiar with the doctrines of the great Hippocrates.

---

A MANUAL OF PRACTICAL THERAPEUTICS—considered with reference to articles of the Materia Medica. By Edward J. Waring, C. I. E., M. D., Fellow of the Royal College of Physicians, London; Surgeon Major (Retired) of her Majesty's Indian army. Edited by Dudley W. Buxton, M. D., B. S., London, member R. C. of Physicians, etc. Fourth edition. Philadelphia: P. Blakiston, Son & Co. 1886. Price—cloth, $3; sheep, $3.50.

The first edition of this standard work was issued thirty-two years ago, since which time therapeutics, both in theory and practice, has undergone many important changes, and has advanced with equal pace with other branches of medical science. The progress has been set forth in each successive edition of the work, bringing it up to the very hour of going to press on each edition; and so with this, the fourth volume; it embraces the very latest discoveries and adopted views on therapeutics. Hence much matter contained in the earlier editions has been greatly curtailed, and many subjects greatly modified, thus making room for the more important new remedies, which are fully treated of. The work is issued in the usual attractive style of the Blakistons.

---

A Compend of Pharmacy. By F. E. Stewart, M. D., Ph. G. Quiz Master in chemistry and theoretical pharmacy in the Philadelphia College of Pharmacy (Alumni Quiz), Lecturer on and Demonstrator of Pharmacy in the Woman's Medical College of Pennsylvania, member of the American Pharmaceutical Association, etc. Based upon Prof. Joseph P. Remington's "Text-book of Pharmacy." Philadelphia: 1886. P. Blakiston, Son & Co., 4012 Walnut street· Cloth: pp. 196.

A very neatly arranged and thorough little book, designed to aid the student in refreshing his memory for the green room. A perusal of its contents will well repay one—any one—for the trouble, for there is a world of "knowledge"—in a very small space. (We *won't* say *multum in parvo.*) It is useful to druggists also. Gotten up in exquisite taste and style by the famous publishers—the Blakistons.

---

Insanity and Its Treatment. Lectures on the treatment, medical and legal; of insane patients. By G. Fielding Blandford, M. D., Oxon, Fellow Royal College of Physicians, London, etc. Third edition, together with *Types of Insanity*, an illustrated guide in the physical diagnosis of mental diseases, by *Allan NcLane Hamilton, M. D.*; one of the consulting physicians to the Insane Asylum of New York city and the Hudson River State Hospital for the Insane, etc. New York: Wm. Wood & Co. 1886.

This is the February number of Wood's library of standard medical authors, uniform in maroon cloth and gold with the series. This new edition, the author says, has been revised by the light of experience, which has enabled him "to estimate the value of the knowledge we possess, to test our remedies and to modify our treatment," in the therapeutics of insanity; and that the discoveries and advances in the physiology and therapeutics of insanity during the last few years have not been of so great importance as those recorded in the former editions.

MEDICAL AND SURGICAL DIRECTORY OF THE UNITED STATES.— The indefatigable publishing house of R. L. Polk & Co., of Detroit, have at last finished the gigantic task of enrolling the physicians and surgeons of America, and have given to the profession and the public one of the most valuable aids to business of nearly every kind, that can be imagined. It is very complete, and contains a world of information, useful not alone to Doctors, but to all business men. It is a catalogue of nearly 80,000 practitioners of Medicine, arranged by *States* and alphabetically; giving the date and place of graduation of all whose data could be obtained, (and here we will say – the means employed to that end were thorough and reliable; experienced, intelligent canvassers were sent into every nook and corner, (for Doctors are found everywhere) and they left nothing undone, to make the Directory complete.) The "kind of Doctor" is indicated by the letter R—for Regular, and H for Homœopathic (or irregular—same thing.) The post office address of each is given, the population of the town or city; together with its location, geographically; all the existing and extinct Medical Colleges in the United States and Canada are put down, with their officers, number of professors, lecturers, demonstrators, etc. together with all the various Medical organizations, from the International Medical Congress and the American Medical Association down to the smallest cross road Medical Society, and even, we believe, "the" Association of so called American Physicians—limited. All the Hospitals, Sanitariums, Asylums and other Medical Institutions are also put down, with their respective governments. The Boards of Health, Town, County and State, and even the National; a synopsis of the laws of registration and other laws relating to the profession in each State, with the names of the proper persons to write to if you happen to have any business in that line; or want to know what little Mesrss. Polk & Co. have not told you about anything and everything that concerns a doctor, in any State, or anywhere; and lastly, the names of all the Medical Journals and other Medical publications, are given, with the names of the editors and publishers, date of publication, subscription price etc. No, not last, for we see here they go on and tell us all about the official lists of officers of the Medical departments of the United States Army, Navy, and

Marine Hospital Service and Roster of examining surgeons of the U. S. Pension Department.

We thought we were surely done, after enumerating all the above; but we find the half has not been told of this wonder of painstaking compilation; for—in the one volume, after one gets tired of lists of names, etc., he can rest his eye by reading descriptive sketches of each State and Territory, giving location, boundary, extent of area (miles and acres), latitude and longitude, statistics relating to climate, temperature, rate of mortality, number of deaths from consumption, etc., and even the names and location of all the best known mineral springs and wells.

If this is not an encyclopœdia of useful knowldege, it would be hard to get up one. The work is turned out in a substantial cloth binding and red edges; is gotten up in good style, and is sold at the very moderate price of *seven dollars* a volume. We would not part with our copy for seven times seven dollars, unless we were sure of being able to replace it.

The public and the profession owe R. L. Polk & Co. a heavy debt of gratitude for this—the most *useful* book that has appeared in many years. In ordering a copy—which we advise you to do, if you have anything to say to physicians, or want to know anything about them, or where they live, what they do, etc., mention to Polk & Co., that you heard of the *Directory* through DANIEL'S *Texas Medical Journal*—if you please.

